# Structural Analysis of Alkaline β-Mannanase from Alkaliphilic *Bacillus* sp. N16-5: Implications for Adaptation to Alkaline Conditions

**DOI:** 10.1371/journal.pone.0014608

**Published:** 2011-01-28

**Authors:** Yueju Zhao, Yunhua Zhang, Yang Cao, Jianxun Qi, Liangwei Mao, Yanfen Xue, Feng Gao, Hao Peng, Xiaowei Wang, George F. Gao, Yanhe Ma

**Affiliations:** 1 State Key Laboratory of Microbial Resources, Institute of Microbiology, Chinese Academy of Sciences, Beijing, People's Republic of China; 2 Graduate University, Chinese Academy of Sciences, Beijing, People's Republic of China; 3 National Laboratory of Biomacromolecules, Institute of Biophysics, Chinese Academy of Sciences, Beijing, People's Republic of China; 4 Key Laboratory of Pathogenic Microbiology and Immunology, Institute of Microbiology, Chinese Academy of Sciences, Beijing, People's Republic of China; 5 College of Life Science, Hubei University, Wuhan, People's Republic of China; New England Biolabs, Inc, United States of America

## Abstract

Significant progress has been made in isolating novel alkaline β-mannanases, however, there is a paucity of information concerning the structural basis for alkaline tolerance displayed by these β-mannanases. We report the catalytic domain structure of an industrially important β-mannanase from the alkaliphilic *Bacillus* sp. N16-5 (BSP165 MAN) at a resolution of 1.6 Å. This enzyme, classified into subfamily 8 in glycosyl hydrolase family 5 (GH5), has a pH optimum of enzymatic activity at pH 9.5 and folds into a classic (β/α)_8_-barrel. In order to gain insight into molecular features for alkaline adaptation, we compared BSP165 MAN with previously reported GH5 β-mannanases. It was revealed that BSP165 MAN and other subfamily 8 β-mannanases have significantly increased hydrophobic and Arg residues content and decreased polar residues, comparing to β-mannanases of subfamily 7 or 10 in GH5 which display optimum activities at lower pH. Further, extensive structural comparisons show alkaline β-mannanases possess a set of distinctive features. Position and length of some helices, strands and loops of the TIM barrel structures are changed, which contributes, to a certain degree, to the distinctly different shaped (β/α)_8_-barrels, thus affecting the catalytic environment of these enzymes. The number of negatively charged residues is increased on the molecular surface, and fewer polar residues are exposed to the solvent. Two amino acid substitutions in the vicinity of the acid/base catalyst were proposed to be possibly responsible for the variation in pH optimum of these homologous enzymes in subfamily 8 of GH5, identified by sequence homology analysis and p*K*_a_ calculations of the active site residues. Mutational analysis has proved that Gln91 and Glu226 are important for BSP165 MAN to function at high pH. These findings are proposed to be possible factors implicated in the alkaline adaptation of GH5 β-mannanases and will help to further understanding of alkaline adaptation mechanism.

## Introduction

Alkaline enzymes show great advantages of functioning under alkaline conditions during the industrial application process as biotransformation catalysts and offer an attractive opportunity for investigating adaptation mechanisms in extreme high pH conditions. Significant progress has been made in isolating novel alkaline enzymes[1], [2], [3], however, their underlying alkaline adaptation mechanisms have not been studied sufficiently.

Beta-mannanases (EC 3.2.1.78) randomly hydrolyze the β-1,4-mannosidic linkages in mannan and heteromannan[4], [5] and these enzymes have great potential uses in the food, paper and detergent industries[6], [7]. Alkaline β-mannanases are capable of functioning under the alkaline conditions during the manufacture of kraft paper and in the detergent industry. Therefore, the discovery of β-mannanases that are stable at alkaline pH has aroused industrial interest. Several alkaline β-mannanases from the alkaliphilic microorganisms *Bacillus* sp. strains AM001, JAMB602, JAMB750, I633 and N16-5, *Bacillus circulans* strains CGMCC1554 and CGMCC1416 and *Bacillus agaradhaerens*, have been characterized and classified into glycosyl hydrolase (GH) families 5 and 26[8], [9], [10], [11], [12], [13], [14]. In addition, the tertiary structures have been solved for β-mannanases from alkaliphilic *Bacillus* sp. JAMB602 (PDB code 1WKY)[10] and *Bacillus agaradhaerens* (PDB code 2WHJ)[15]. However, there is a paucity of information concerning the structural basis for the alkaline tolerance displayed by β-mannanases.

Of all the characterized alkaline β-mannanases, the one from *Bacillus* sp. strain N16-5 (BSP165 MAN) has stimulated great interest. This enzyme not only shows a pH optimum as high as 9.5 but also has a high specific activity of 5065 U/mg. These properties, combined with its characteristics of stability under alkaline conditions and insensitivity to some surfactants, would qualify the mannanase as a candidate in the manufacture of kraft pulp and in the detergent industry. The catalytic domain of BSP165 MAN, which shows virtually the same pH activity profile and specific activity as the full-length enzyme, has been expressed, purified and crystallized and preliminary X-ray studies have been performed[16]. Solving the three-dimensional structure of BSP165 MAN would help to decipher the structural basis for alkaline adaptation of the GH5 β-mannanases.

Here, we report the crystal structure of the catalytic domain of BSP165 MAN. To investigate the distinct structural features linked to alkaline adaptation, we carried out a comprehensive comparative analysis of BSP165 MAN with other family 5 mannanases. This is the first report to investigate the basis of alkaline adaptation of β-mannanases. These findings will not only deepen our understanding of adaptation mechanism of proteins to alkaline conditions, but will also help in the optimization of the pH-dependent characteristics of enzymes for a broad range of industrial applications.

## Results

### Structure determination of the β-mannanase from Bacillus sp. strain N16-5

The crystal structure of the catalytic domain of BSP165 MAN has been determined at a resolution of 1.6 Å by molecular replacement using the coordinates of alkaliphilic *Bacillus* sp. JAMB-602 β-mannanase (PDB code, 1WKY; sequence identity, 78%)[10], which also belongs to the GH subfamily 5–8, as a search model. Refinement of BSP165 MAN at a resolution of 1.6 Å resulted in an electron density map of excellent quality for the majority of the residues. However, weak electron density was obtained for a number of highly mobile side chains on the solvent exposed molecular surface. Residues 260 and 261 were not modeled due to lack of sufficient electron density data and, hence, are not included in the refinement. Asp65 has two possible conformations and the side chain of Asp65 seems to have considerable mobility based on the electron density map.

Towards the end of the refinement, water molecules were evident in the electron density maps and were included in the model. One peak of tetrahedral geometry was interpreted as SO_4_^2–^, based on the 2Fo-Fc map contoured at 2 σ and their presence in the crystallization medium.

The final model, consisting of 295 amino acid residues, has a crystallographic R-factor of 17.4% and an R-free value of 21.5%. A Ramachandran plot confirmed the stereochemical validity where all residues are in allowed regions with acceptable values of bond angle geometry. Details of the refinement and stereochemistry of the final model are provided in Table 1.

**Table 1:** Crystal parameters and refinement quality statistics.

Parameters	BSP165 MAN
Crystallographic data	
Space group	P2_1_2_1_2_1_
Cell parameters(Å, °)	a = 59.0, b = 63.3, c = 83.3,α = β = γ = 90
Data collection	
Resolution range (Å)	63.29 – 1.60 (1.71– 1.60)
Total number of reflections	182424(7471)
Number of unique reflections	38945(4841)
Average redundancy	4.68 (1.54)
Completeness (%)	92.74 (65.44)
R_sym_ (%)^a^	7.6 (40.7)
Average *I/σ* (*I*)	7.63 (1.95)
Refinement	
R_factor_/R_free_ (%)	17.4/21.5
No. of all protein atoms	2315
Mean B-values (Å^2^)	6.9
No. of SO_4_ atoms	5
Mean B-values (Å^2^)	17.5
No. of water molecules	579
Mean B-values (Å^2^)	25.3
RMSD bonds (Å^2^)	0.007
RMSD angles (°)	1.1
Ramachandran Plot %-residue in the most favored region b	89.5%
%-residue in additional allowed region	10.2%
%-residue in generously allowed region	0.4%
%-residue in disallowed region	0
PDB code	3JUG

Values in parentheses correspond to the highest resolution shell.

a *R*_sym_  =  ∑**_h_** ∑*_i_* |*I* **_h_**_*i*_ -〈*I* **_h_**〉|/∑**_h_** ∑*_i_* 〈*I* **_h_**〉, where *I* **_h_**_*i*_ is the *i*th observation of reflection **h** and〈*I* **_h_**〉is the weighted average intensity for all observations *i* of reflection **h**.

b Calculated for nonglycine and nonproline residues using PROCHECK.

### Overall structure of the β-mannanase from Bacillus sp. strain N16-5

**Figure 1:**
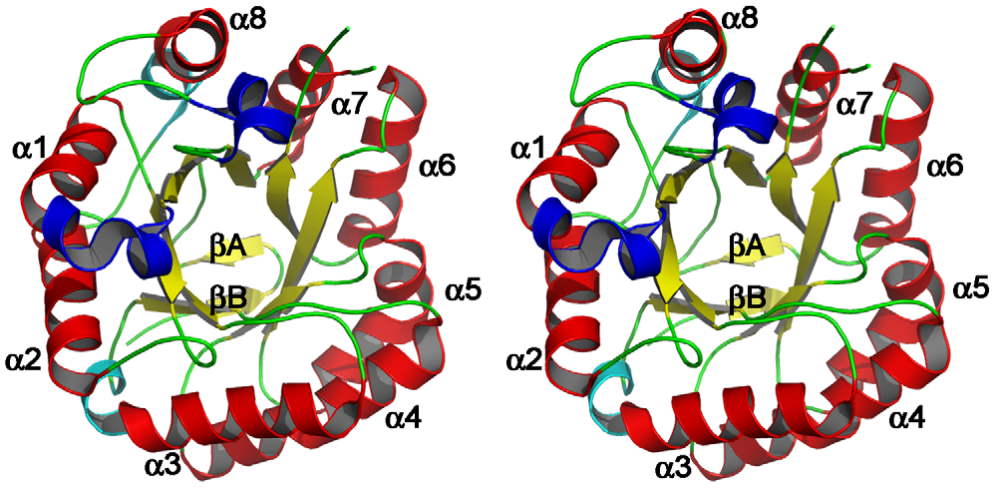
Overall structure of BSP165 MAN from the top view. Major α-helices and β-strands are drawn in red and yellow, respectively, and labeled according to the ideal (β/α)_8_ barrel structure. Two short β-strands at the N terminus are colored in yellow, and additional two α-helices and three 3_10_-helices are in cyan and blue, respectively.

The crystal structure of the catalytic domain of BSP165 MAN adopts the canonical (β/α)_8_-barrel architecture. It is composed of ten β-strands and thirteen α-helices including three 3_10_-helices (residue 60–62, 64–66 and 289–294). The β-sheet structures consisting of eight parallel β-strands (yellow) lie in the middle forming a barrel surrounded by eight α-helices (red) with a size of 45×45×40 Å^3^. Aside from the structural elements of the barrel, two short β-strands (yellow) at the N terminus form the bottom of the barrel (Figure 1). Additional secondary structures including two α-helices (cyan) and three 3_10_-helices (blue) are on the bottom and top of barrel, respectively.

### Comparison with other Family 5 β-mannanases

Amino acid sequences of 30 family 5 β-mannanases reported in literature with known pH-dependent activity were retrieved. Besides structure of BSP165 MAN, 3D structures have been solved for other 5 enzymes (BA MAN, *Bacillus agaradhaerens* β-mannanase; BSP602 MAN, *Bacillus* sp. JAMB-602 β-mannanase; TF MAN, *Thermomonospora fusca* β-mannanase; ME MAN, *Mytilus edulis* β-mannanase; HJ MAN, *Hypocrea jecorina* RUTC-30 β-mannanase in Table S1). The molecular phylogeny of these enzymes is shown in Figure S1. Major branches form two clusters: cluster A which is composed of 13 bacterial β-mannanases classified into subfamily 5–8 and cluster B which includes 17 β-mannanases mainly from eukaryota, classified into subfamily 5–7 and 5–10. Alkaline mannanases, BSP165 MAN and its close homologues (BA, BSP602, BC15554 and BC1416 MAN), all belong to subfamily 5–8 in cluster A, while most of mannanases of cluster B act at acidic pH.

### Total amino acid composition of family 5 β-mannanases

A thorough comparison of enzymes from cluster A (classified into GH5-8) with those from cluster B (grouped into GH5-7 and GH5-10) was carried out (Table 2). The differences were assessed with the Student's t-value (a negative sign was added to the value if the average is smaller for GH5-8 enzymes). Distinct difference in pH optimum was observed, indicating that β-mannanases of GH5-8 are active at higher pH than those of GH5-7 and GH5-10.

**Table 2:** Molecular features comparison of family 5 β-mannanases grouped into different phylogenetic clusters.

Parameters	Mean		t-value	*P*
	Cluster A	Cluster B		
**pH**	**7.365**	**5.0441**	**4.274**	**0.000**
pI_calc_	5.0577	5.3047	−0.784	0.439
**Mw**	**27.7738**	**33.8341**	**−3.800**	**0.001**
Ala (%)	9.6762	8.2924	2.561	0.16
**Cys (%)**	**0.3392**	**1.1541**	**−3.563**	**0.001**
Asp (%)	7.4938	6.4871	1.381	0.178
Glu (%)	4.7523	4.2018	1.052	0.302
**Phe (%)**	**2.4531**	**3.8906**	**−5.025**	**0.000**
Gly (%)	9.3115	9.4806	**−**0.332	0.742
His (%)	2.6131	2.4888	0.349	0.730
**Ile (%)**	**7.1185**	**5.2129**	**4.330**	**0.000**
Lys (%)	3.9623	4.9459	**−**1.291	0.212
Leu (%)	6.0323	6.4294	**−**0.867	0.393
Met (%)	1.9377	2.0371	**−**0.314	0.756
Asn (%)	7.8969	6.8153	1.650	0.110
**Pro (%)**	**2.4338**	**3.1929**	**−2.424**	**0.022**
Gln (%)	3.2785	3.5076	**−**0.611	0.546
**Arg (%)**	**3.4154**	**2.1576**	**2.846**	**0.008**
Ser (%)	6.5146	7.2935	**−**0.977	0.337
**Thr** **(%)**	**5.3208**	**7.1341**	**−3.271**	**0.003**
**Val** **(%)**	**7.2915**	**6.0935**	**3.094**	**0.004**
Trp (%)	3.7269	3.5029	1.096	0.282
**Tyr** **(%)**	**4.4315**	**5.6829**	**−3.658**	**0.001**
Charged (RKHYCDE) **(%)**	27.0077	27.1182	**−**0.072	0.943
Acidic (DE) (%)	12.2462	10.6888	1.593	0.122
Basic (KR) (%)	7.3777	7.1035	0.403	0.690
**Polar (NCQSTY) (%)**	**27.7815**	**31.5876**	**−2.725**	**0.011**
**Hydrophobic (AILFWV)** **(%)**	**36.2985**	**33.4218**	**4.012**	**0.000**
**Number of total amino acid residues**	**251.4615**	**306.7647**	**−3.903**	**0.001**

Alteration of amino acid composition is considered to relate to protein adaptation to extreme environments, so we further compared the amino acid compositions within catalytic domains of enzymes from the two clusters. The most significant differences between the two groups were the increase in hydrophobic residues and Ile contents, and decrease in Phe contents (amino acid contents-Hydrophobic, Ile and Phe in Table 2, *P* = 0.000, respectively). Reductions in polar residues, Cys, Pro, Thr and Tyr contents and increase in Arg and Val contents were also observed as significant events (amino acid contents-Polar, Cys, Pro, Thr, Tyr, Arg and Val in Table 2, *P* = 0.011, 0.001, 0.022, 0.003, 0.001, 0.008 and 0.004). These findings indicate increasing the relative frequency of hydrophobic residues and Arg residue as well as decreasing that of polar residues could relate to an increase in the optimum pH for the enzyme activity.

### Structure comparison of characterized Family 5 β-mannanases

In order to understand the structural basis for alkaline stability, the tertiary structures of BSP165 MAN (PDB code 3jug, optimum pH 9.5) and its close homologues, BA (PDB code 2whj, optimum pH 8–10)[15] and BSP602 MAN (PDB code 1wky, optimum pH 9), have been compared with those of three other family 5 β-mannanases with known 3D structures and characterized to be active at neutral or acidic pH (TF MAN, PDB code 1bqc, optimum pH 6–8[17]; ME MAN, PDB code 2c0h, optimum pH 5.2[18], [19]; HJ MAN, PDB code 1qno, optimum pH 3.5–4[20], [21]) (Table S1).

### Secondary structure

**Figure 2:**
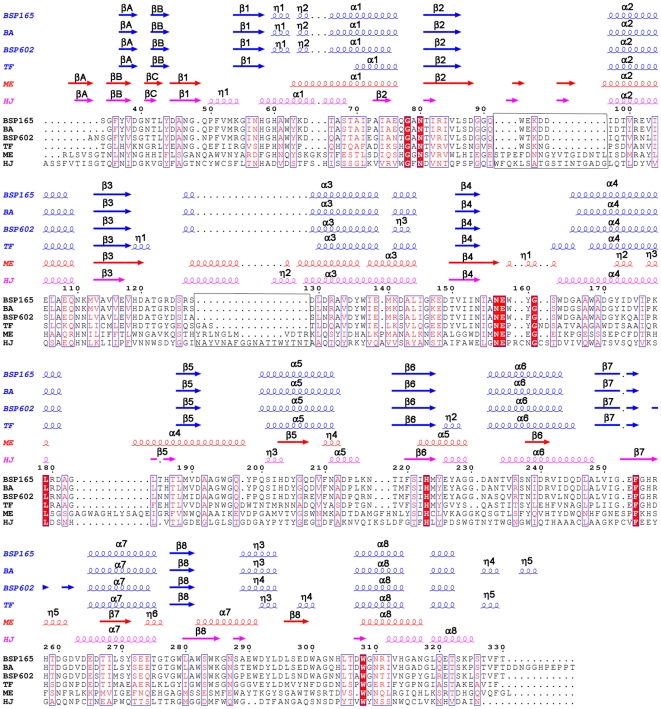
Structural alignment of mannanases from *Bacillus* sp. N16-5 (BSP165), *Bacillus agaradhaerens* (BA), *Bacillus* sp. *strain* JAMB-602 (BSP602), *Thermobifida fusca* KW3 (TF), *Hypocrea jecorina* RUTC-30 (HJ) and *Mytilus edulis* (ME). The residues are numbered as in the BSP165 MAN model. Secondary structure elements for BSP165, BA, BSP602 and TF are presented in blue, while those for HJ and ME are colored in red and magenta, respectively. The figure was produced using ESPript[55].

The secondary structure compositions are similar in all of the six GH5 family β-mannanases (Table S2). Figure 2 reveals that the secondary structures of the other two alkaline β-mannanases (BA and BSP602 MAN) are the most similar to that of BSP165 MAN, followed by neutral TF MAN and acidic enzymes (ME and HJ MAN).

Compared with the neutrophilic TF MAN enzyme, the alkaline β-mannanases (BSP165, BA and BSP602 MAN) show slight differences in secondary structures. Residues from the randomly coiled loop connecting β1α1 in TF MAN form two 3_10_ helices in alkaline β-mannanases. Loops 119–121, 162–165 and 226–228 (according to the sequence of BSP165 MAN) in these alkaline enzymes are substituted by short α-helices (residues 86–88, 134–137 and 199–201) at the corresponding regions in TF MAN.

Comparing to the ME MAN and HJ MAN, alkaline and neutral β-mannanases have two but not three β-strands at the N-terminal end of the sequence forming a motif to cap the barrel, and the loops connecting the αβ-repeats are, in general, shorter with less extra short β-strands and 3_10_-helices formed. Besides, they show obvious differences in the length of the main secondary structural elements.

Altogether, different shapes of TIM-barrels are presented (Figure S2).

### Comparison of hydrogen bonds and ion pairs

In this study, changes in the number of ion pairs and hydrogen bonds were detected in the β-mannanases and the relationship between these changes and alkaline adaptation was investigated (Table S2). Similar content of ion pairs and hydrogen bonds were observed, indicating their changes may not involve in alkaline adaptation of these GH5 β-mannanases.

### Protein surface comparison

Changes in amino acid content on the solvent-accessible surface were detected to check whether any correlation with alkaline adaptation can be found in these six β-mannanases. The results are presented in Table 3. The percentage of solvent-exposed negatively charged residues (Asp and Glu) is higher for alkaline-stable β-mannanases than for neutrophilic and acid-stable β-mannanases, which results in a higher ratio of negatively to positively charged residues. At the same time, the percentage of polar residues (Asn, Gln, His, Thr and Ser), especially that of easily-decomposed residues (Ser and Thr), is reduced in alkaline β-mannanases. Besides, BSP165 and BA MAN contain lower number of Asn and Gln residues that are alkaline-susceptible (Asn+Gln in Table 3, 14.3% and 14.5%, respectively), when compared with the other four mannanases active at lower pH (Asn+Gln for BSP602, TF, ME and HJ MAN in Table 3, 23.2%, 24.5%, 23.8% and 32.4%, respectively).

**Table 3:** Amino acid content on the surface of GH5 β-mannanases with known 3D structure.

Parameters	BSP165	BA	BSP602	TF	ME	HJ
PDB code	3jug	2whj	1wky	1bqc	2c0h	1qno
Total	56	55	56	53	63	74
Asp (%)	23.2	25.5	14.3	7.5	4.8	5.4
Glu (%)	17.9	18.2	10.7	7.5	3.2	1.4
Negatively charged (%)	41.1	43.6	25	15.1	7.91	6.8
Arg (%)	7.1	1.8	12.5	1.9	7.94	1.4
Lys (%)	7.1	7.3	3.6	7.5	11.1	6.8
Positively charged (%)	14.3	9.1	16.1	9.41	19.0	8.1
Negatively charged/positively charged (%)	2.9	4.8	1.6	1.6	0.4	0.8
Asn (%)	7.1	5.5	17.9	11.3	17.5	13.5
Gln (%)	7.1	9.1	5.4	13.2	6.3	18.9
His (%)	3.6	3.6	0	0	7.9	1.4
Thr (%)	1.8	7.3	3.6	3.8	4.8	16.2
Ser (%)	5.4	5.5	8.9	15.1	15.9	14.9
Polar, uncharged (%)	25	30.9	35.7	43.4	52.4	64.9
Tyr (%)	3.6	1.8	1.8	5.7	1.6	1.4
Gly (%)	0	1.8	1.8	3.8	7.9	8.1
Pro (%)	5.4	5.5	8.9	7.5	1.6	2.7
Trp (%)	5.4	5.5	1.8	1.9	3.2	0
Leu (%)	0	0	1.8	0	0	0
Ile (%)	1.8	0	1.8	0	0	0
Val (%)	0	0	0	0	3.2	0
Ala (%)	3.6	1.8	5.4	13.2	3.2	8.1
Hydrophobic (%)	19.6	16.4	23.2	32.1	20.6	20.3
Asn+Gln (%)	14.3	14.5	23.2	24.5	23.8	32.4
Thr+Ser (%)	7.2	12.8	12.5	18.9	20.7	31.1

### Active site comparison

Similar to all other studied GH5 β-mannanases, the proton donor (Glu 158) in BSP165 MAN is located at the C-terminus of β4 and the nucleophile (Glu 253) is located at the C-terminus of β7[11]. Six other amino acids (Arg83, His119, Asn157, His223, Tyr225 and Trp282), located near the catalytic centre, are highly conserved in all the four family 5–8 β-mannanases (Figure S3A). Except His119, seven of the eight conserved residues are preserved in ME and HJ MAN (Figure S3, B and C).

Four structure-solved GH5-8 β-mannanases catalyze mannan under quite different pH conditions, despite sharing high sequence and structural identity. Amino acid residues within radius of 12 Å from either of the two catalytic amino acids in BSP165, BA, BSP602 and TF MAN were assessed to identify residues that play an important role in determining the pH optima of these homologous β-mannanases. The majority of the residues surrounding Glu158 and Glu253 are absolutely conserved, with the exception of several residues.

**Figure 3:**
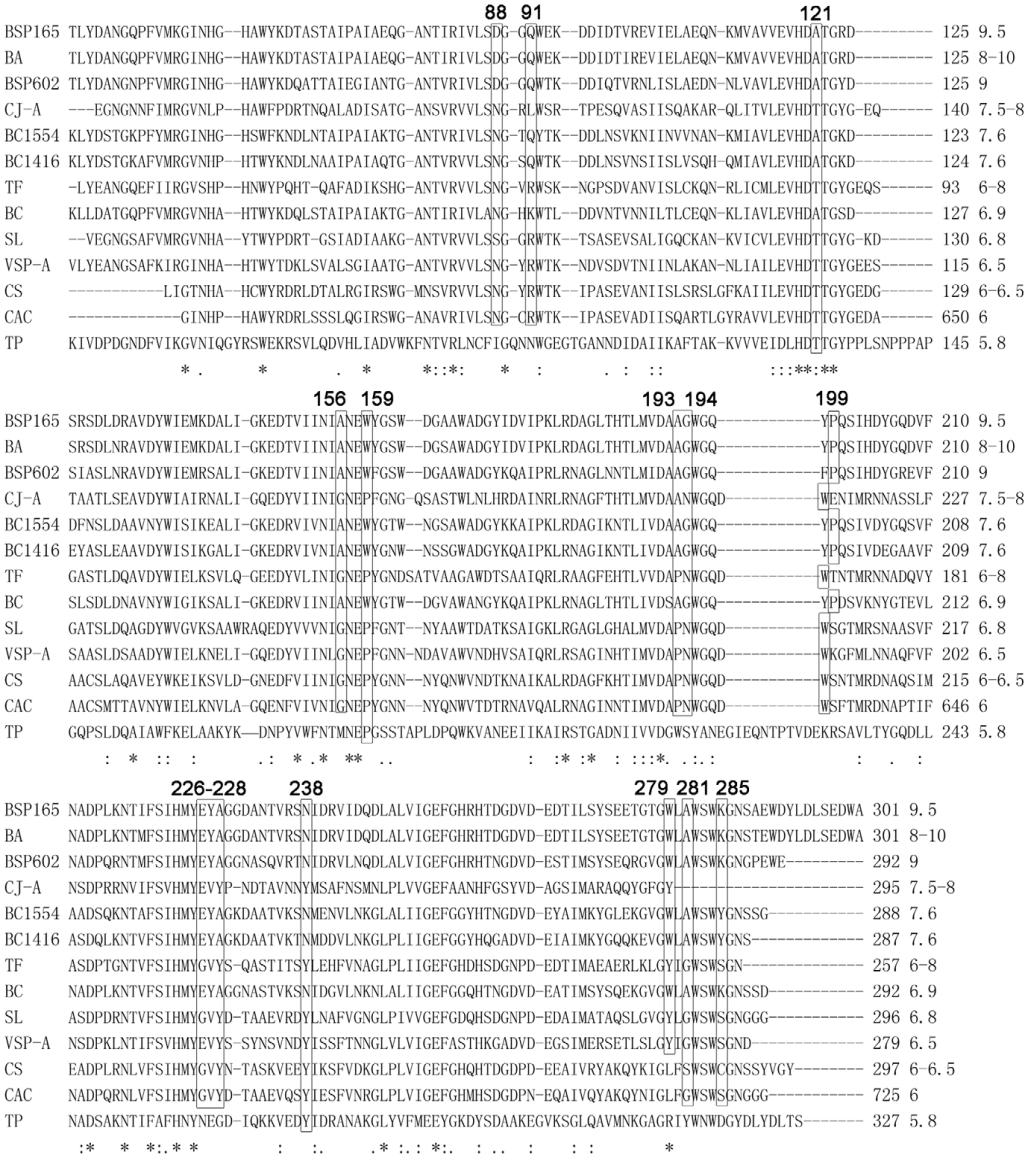
Multiple sequence alignment of GH5-8 mannanases that pH-dependent activities have been characterized. Amino acid residues possibly responsible for the different pH-related activities are labeled. The numbers following the amino acid number indicate the pH optimum.

The sequences of all of the characterized β-mannanases in the GH5-8 family were aligned to illustrate the function of these different sites mentioned above in regulating the pH optima of β-mannanases, and 15 residues were supposed to be determinants of catalysis under different pH conditions (Figure 3). To evaluate this hypothesis, structures of reciprocal single-site mutants at these 15 positions around the active site of BSP165 and TF MAN in complex with mannotriose were constructed and p*K*_a_ calculations were carried out (Table 4). We define mutations that would be discernible in the pH-activity profile as mutations that give a shift of more than 0.3 p*K*_a_ units for either of the two catalytic acids. The results showed that mutations at positions corresponding to Gln-91 and Glu-226 of BSP165 MAN should give p*K*_a_ shifts that would be discernible in the pH-activity profiles for both enzymes in opposite directions as predicted. Therefore, we supposed that these two sites possibly played an important role in regulating pH optima of these homologues.

**Table 4:** Calculated changes of p*K*_a_ values and pH optima of the mutant BSP165 and TF MAN.

Mutant		Δp*K*_a_ (proton donor)		Δp*K*_a_ (catalytic base)		ΔpH_opt_	
BSP165	TF	Δp*K*_a_ (E158)	Δp*K*_a_ (E128)	Δp*K*_a_ (E253)	Δp*K*_a_ (E225)	BSP165	TF
D88N	N55D	−0.159	0.039	0.018	−0.011	−0.07	0.014
**Q91R**	**R58Q**	−**0.464**	**0.542**	−**0.411**	**0.437**	−**0.438**	**0.4895**
A121T	T88A	0.005	0.152	0.025	0.082	0.015	0.117
A156G	G126A	−0.088	−0.001	0.026	−0.033	−0.031	−0.017
W159P	P129W	−0.114	0.099	−0.019	0	−0.066	0.0495
A193P	P165A	−0.086	0.053	−0.071	0.014	−0.079	0.0335
G194N	N166G	−0.184	0.044	−0.071	0.061	−0.128	0.0525
P199W	W171P	−0.014	0.038	−0.038	0.081	−0.026	0.0595
**E226G**	**G199E**	−**0.898**	**0.378**	−0.049	−0.055	−**0.474**	**0.1615**
Y227V	V200Y	−0.061	0.022	−0.1	−0.01	−0.081	0.006
A228Y	Y201A	−0.058	−0.007	−0.008	0.084	−0.033	0.0385
N238Y	Y210N	−0.068	−0.048	−0.094	−0.051	−0.081	−0.0495
W279Y	Y251W	−0.047	0.01	0.094	0.017	0.0235	0.0135
A281G	G253A	−0.013	0.006	−0.003	−0.031	−0.008	−0.0125
K285S	S257K	−0.016	−0.043	0.054	−0.113	0.019	−0.078

**Figure 4:**
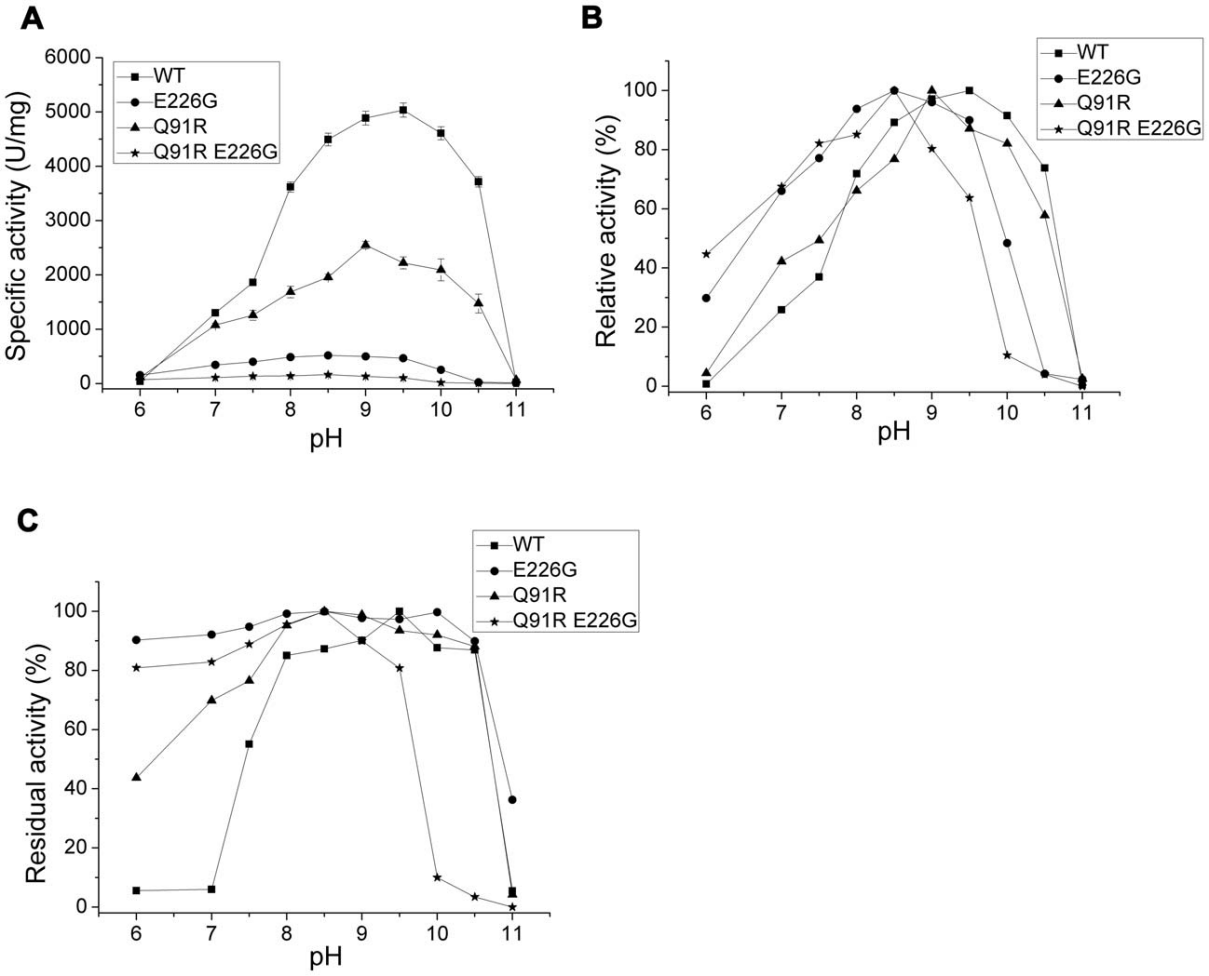
Effect of pH on the activity and stability of wild type and the mutant BS165 MAN. A: pH-dependent specific activity. B: pH-dependent relative activity. C: pH stability. Wild type (▪), Q91R (▴), E226G (•) and Q91R E226G (★).

Further, we constructed 2 single (Q91R and E226G) and one double (QE to RG at position 91 and 226) point mutations to check their influence on pH regulation for BSP165 MAN. The pH-activity profiles are shown in Figure 4. Q91R decreases the activity of BSP165 MAN to about 51% of the wild-type activity and its pH-activity profile is pushed slightly towards more acidic pH values. pH-activity profiles for E226G and the double mutant have become almost flat and been shifted to more acidic pH values with optimum pH 8.5, which was lower by one unit than the optimum pH for the activity of the wild type mannanases.

The double mutant exhibited significantly reduced alkaline stability, while all the mutants had strengthened stability at pH 6–7.5 when compared to the wild-type enzyme (Figure 4C).

## Discussion

Our target enzyme alkaline BSP165 MAN showed pH optimum as high as 9.5, one of the highest among all the known characterized GH5 mannanases. All these enzymes could be divided into clusters A and B based on the phylogenetic tree. Coincidentally, they displayed significantly different pH optimum.

### Amino acid composition comparison

Based on previous studies, amino acid composition is considered to be correlated to protein adaptation to extreme environments. Several interesting studies have been reported on the relation of amino acid content and alkaline adaptation of enzymes[22], [23], [24], [25]. For alkaline proteases and cellulases, a decrease in the number of Asp and Lys residues and an increase in Arg, His and Gln residues were observed during the alkaline adaptation process[22], [23]. To explore the underlying mechanism of alkaline adaptation of GH5 mannanases, we analyzed the differences of amino acid composition among these two clusters.

The consensus amino acid content changes for the alkaline adaptation of proteases, cellulases and GH5 mannanases was an increase in the Arg content, whose p*K*_a_ value is generally higher, considered helpful for maintaining the local charge balance at higher pH[23].

Besides, GH5 mannanases have their own specific strategy to adapt to alkaline conditions by regulating amino acid contents. Mannanases in cluster A which are active at higher pH contain lower total number of polar residues, especially lower number of Tyr, Thr (easily decomposed) and Cys (easily oxidized), and that would result in a better stability under alkaline environment (Table 2). Meanwhile, an increase of hydrophobic residues (Ala, Ile, Leu, Phe, Trp and Val) was observed, and that would help cluster A mannanases to form more compact protein structures. We further calculated the compactness of the six structure-known mannanases active at quite different pH conditions, shown in table S2, and found a negative correlation between compactness coefficient and the optimum pH for enzyme activity (Pearson correlation coefficient = −0.906, *P* = 0.013). It indicates a trend towards increase in compactness with an increase in optimum pH. Thus, increased compactness may contribute to structural adaptation of mannanases to alkaline pH. No obvious differences in the number of lysine residues between these two clusters of β-mannanases were observed, although loss of lysine residues during the alkaline adaptation process of amylases, cellulases and phosphoserine aminotransferases has been observed[24]. These observations suggest that changes in the composition of amino acid residues as mechanism of alkaline adaptation might not be universal but specific for each protein.

### Secondary structures

Adaptation to extreme conditions can occur in evolution within a short time in both directions by incorporation or exchange of short sequences with the appropriate physical-chemical properties without the need for gradual changes over the whole polypeptide chain[26]. When the six structure-known enzymes were aligned, two major inserted stretches >10 amino acids (Figure 2) were observed in acidic mannanases. The percentages of polar residues (easily decomposed Ser and Thr, easily oxidized Cys, alkaline susceptible Asn and Gln) in the two inserted stretches in acidic mannanases (ME and HJ MAN) are 34.5% and 44.4% respectively, much higher than that in the neutral and alkaline enzymes (29.7% in TF MAN, 24.6% in BA MAN, 27.5% in BSP602 MAN and 23.5% in BSP165 MAN). Accordingly, it is proposed that deleting stretches rich in polar residues that are easily deaminated and oxidized at alkaline pH to acquire better stability could be a means for rapid alkaline adaptation of mannanases.

Rearrangement of helices, strands and loops in the active regions caused by the secondary structural differences, contributes, to a certain degree, to the distinctly different shaped catalytic clefts in these enzymes (Figure S2). These differences above lead to changes in the polarity, charge distribution and/or hydrogen bonding in the microenvironment of the active site of these β-mannanases, which might in turn affect the protonation state of key catalytic amino acids, and subsequently determine the pH optimum of the enzyme. Changing the microenvironment of the active site by site-directed mutagenesis has been shown to increase the pH optimum of glucoamylase from *Aspergillus awamori* [27]. Thereby we propose that reorganization of the chameleon-like TIM barrel surrounding the catalytic center of the protein could be a mechanism for adaptation to alkaline conditions.

### Hydrogen bonds and ion pairs

Despite numerous previous studies[22], [23], [28], no general strategy has yet been confirmed by which an enzyme can adapt to high pH by changing the numbers of ion pairs. Strategies are generally specific for each enzyme class. In this study, for β-mannanases there is no correlation between changes in the number of ionic interactions and alkaline adaptation.

An increase of hydrogen bonds corresponds to a more compact and rigid protein conformation, which is probably favorable for stabilizing the protein against alkali-denaturation and suggested to be an important mechanism for structural adaptation of a variety of proteins to alkaline environments[22], [28]. However, it appears that in the alkaline β-mannanases an increase in the number of hydrogen bonds might not be necessary for their enhanced resistance to alkaline pH. Perhaps, increased hydrogen bonding is a favorable but non-essential strategy used by some proteins for alkaline tolerance.

### Protein surface

The surface of a protein constitutes the interface through which a protein senses its environment[29]. Extremozymes employ a series of protective strategies to adapt to different extreme conditions, and one of these strategies is changing the content of surface amino acids.

Similar to alkaline xylanases from *Bacillus* sp. N27 and *Bacillus halodurans* S7, BSP165 MAN and its alkaline homologues have more abundant surface-accessible negatively charged residues when compared with the nonalkaline counterparts. Protein surfaces rich in acidic residues probably play an essential role in maintaining protein function under alkaline conditions. Generally, acidic residues exhibit a strong water binding capacity in the deprotonated state at high pH[30] and the presence of numerous negatively charged carboxylates on the surface of a protein can keep the protein completely hydrated, thus preventing it from aggregating[31]. In addition, numerous surface-exposed negatively charged carboxylates might help protect the protein core from OH^−^ attack, stabilizing the protein under alkaline conditions[32].

There are also significant differences in the total number of polar residues on the surface between alkaline and nonalkaline ones, especially in the number of Ser and Thr residues. Since an excess of polar residues on the protein surface might disturb the local stability at higher pH due to their unstable physical-chemical properties, the absence of these residues might help to maintain the balance.

### Active site

In general, the pH-activity profile of retaining glycosyl hydrolases is governed by the p*K*_a_ values of the proton donor and the catalytic nucleophilic residue [33], [34], [35], [36], [37]. Some key residues in the vicinity of the active site, directly or indirectly interacting with the catalytic carboxylates, can affect the p*K*_a_ values of the catalytic residues by changing the electrostatic and/or dynamic aspects of the active site[38], and are at least partially responsible for the different pH-dependent activities of homologous enzymes[39], [40]. Identification of key residues based on comparisons with homologous enzyme that possesses obvious distinct pH-activity profile has been proven effective. Thus, 15 residues within a radius of 12 Å from either of the two catalytic amino acids in BSP165 and TF MAN were selected based on structural and homologous sequences alignment. We calculated the achievable Δp*K*_a_ values for two target titratable groups in BSP165 and TF MAN by introducing reciprocal mutations. The results showed mutations at amino acid sites Q91 and E226 (numbering based on BSP165 MAN) significantly changed p*K*_a_ values in both enzymes to opposite direction as deduced. Further, pH-activity profiles of mutants at position 91 and 226 were analyzed. The direction of the pH-activity profile shifts for BSP165 MAN mutants are reproduced well by the p*K*_a_ calculations. Especially the double mutation at the two sites has shown a substantially decreased activity in the alkaline range (Figure 4). These experimental data have proved these two sites are important for high pH catalysis of BSP165 MAN. This would mean that mutations at amino acid sites Q91 and E226 (numbering based on BSP165 MAN) might be involved in an evolutionarily critical change in the pH optimum for GH5-8 mannanases.

**Figure 5:**
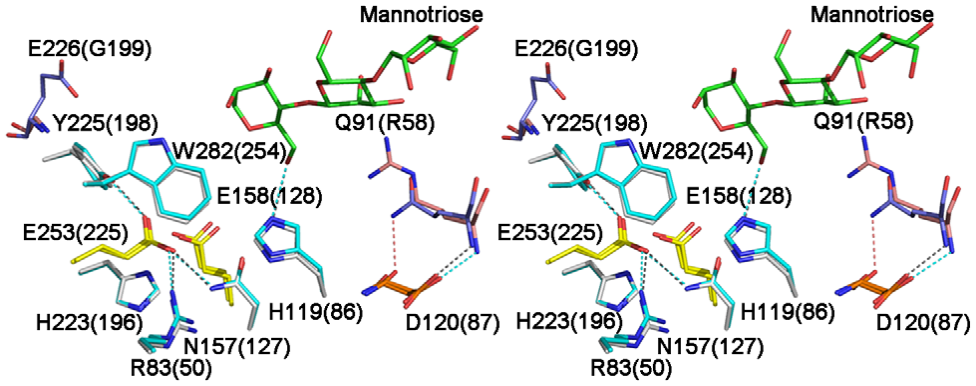
Stereo view of two point mutation sites around the catalytic center in BSP165 MAN (Q91 and E226, shown in blue) and TF MAN (R58 and G199, shown in pink). Hydrogen bonds are represented by dashed lines. Mannotriose bound to TF MAN is shown in green.

Gln91 is situated in a loop connecting β-stand 2 and α-helix 2 of the TIM-barrel. Its distances from the target groups are 11.50 Å (Glu158 Cδ) and 14.06 Å (Glu253 Cδ), respectively (Figure 5). Probably, the mutation Q91R brings the electrostatic changes to the active site by introducing a positive charge and in this way perturbing the p*K*_a_ values of the active site acids. Glu226 sits in the loop that covers the active site and is next to Tyr225, one of the eight conserved amino acids in GH5-8 mannanases (Figure 5). Changes of the solvent accessibility and the dynamics of Tyr225 introduced by the mutation of E226G might perturb the stabilization of the active-site environment and influence the protonation state of the catalytic glutamate residues. We speculate that the pH-activity profile shift is a combined effect of the charge and the change in mobility of Tyr225 that are induced by mutation of E226G. However, the magnitude of the pH-activity profile shifts is not well reproduced, especially for E226G. The discrepancy is not readily explainable and is possibly due to changes in stability.

In conclusion, the crystal structure of the catalytic domain of mannanase from alkaliphilic *Bacillus* sp. N16-5, BS165 MAN, has been determined at 1.6 Å resolution. In order to illustrate molecular mechanism for alkaline adaptation in GH5 β-mannanases, thorough comparisons were carried out and several distinctive structural features were identified. In mannanases active at elevated pH, an increase in the hydrophobic residues and Arg residue content and a decrease in polar residues were observed on the level of primary structure comparison. Besides, changes in the position and length of helices, strands and loops were found, which might contribute to subtle influence on the catalytic environment, thus affecting the pH-dependent properties. Negatively charged residues were favored on the surface of alkaline mannanases, as reported for other alkaline active enzymes, at the expense of polar residues. p*K*_a_ calculations and analysis of mutant mannanases further revealed that the location of Gln91 and Glu226 render BSP165 MAN active under high pH conditions, and mutations at the two sites might be involved in an evolutionarily critical change in the pH optimum for the activity of GH5-8 mannanases. Our analysis would help to engineer proteins with improved stability and activity in alkaline conditions, a requirement for biotechnological applications.

## Materials and Methods

### Crystallization, data collection, structure resolution and refinement

The purification, crystallization and data collection of the catalytic domain of the enzyme were carried out as previously described[16]. Briefly, crystals with average dimensions of 50×100×200 µm were obtained at 25°C in 2–3 days by setting up 2 µl crystallization drops containing 0.5 M (NH_4_)_2_SO_4_, 0.1 M sodium-citrate (pH 5.6), 1.2 M Li_2_SO_4_ and 2.0 mg ml^−1^ protein. The structure was solved by the molecular replacement method with the program MOLREP[41] from the CCP4 program package[42], using the crystal structure of the β-mannanase from alkaliphilic *Bacillus* sp. JAMB-602 (PDB code 1WKY) as the search model. There is one molecule in the asymmetric unit that corresponds to the Matthews coefficient[43] of 2.36 Å^3^Da^–1^. Refinement was carried out using the parameters of Engh and Huber[44] and model building was carried out using Phenix (1.3)[45]. Five percent of randomly selected observed reflections were kept aside for the cross-validation. The stereochemistry of the final models was analyzed with PROCHECK[46], and RMSD resulted in the proper values (Table 1).

### Phylogenetic analysis

The characterized family 5 β-mannanases were selected referring to http://www.cazy.org/GH5_characterized.html. The ClustalW program[47] was used for multiple sequence alignment of amino acid sequences. Sequences encoding their catalytic domains were retrieved from the GenBank database based on the notation in the features. Phylogenetic tree was constructed with the neighbor-joining method[48] and the minimum evolution method[49], both from MEGA software version 3.1[50]. The p-distance and Poisson correction substitution models were used in both tree-building methods. Bootstrap values were calculated based on 1,000 replicates of the data[51].

### Comparison with other family 5 β-mannanases

The total amino acid compositions of the catalytic domains were calculated with MEGA software version 3.1. The two-sample Student's *t*-value was then calculated using PASW statistics 18 (SPSS Inc., Chicago, Illionis).

### Structural analysis

The solvent-exposed residues were identified using the cutoff value of ≥30% for the relative surface accessibility area of the side chain in Swiss-PDB Viewer[52] using the default probe radius. Hydrogen bonds and contacts were assessed by HBPLUS[53]. Secondary structure elements were analyzed with DSSP[54]. Structure-based alignment was performed using ESPript[55]. Molecule figures were created using PyMOL (Delano Scientific LLC, Palo Alto, CA, USA). Compactness coefficient is defined as the ratio of the accessible surface area of a protein to that of the ideal sphere of the same volume[56].

### pK_a_ calculations

The p*K*_a_ of ionizable groups were predicted by using MCCE version 2.4[57], [58], [59]. MCCE uses the DelPhi program version 4[60]. The parameters were set as follows: ionic strength 0.05 M, initial pH −30.0, titration points 60 and other default values.

A model of BSP165 MAN in complex with mannotriose was constructed from the TF Man- mannotriose X-ray structure[17].

### Preparation of mutant structures

Mutant structures for p*K*_a_ calculations were designed using backbone dependent rotamer library[61].

### Mutagenesis and enzyme preparation

Site-directed mutagenesis was performed using the QuikChange Site-Directed Mutagenesis Kit (Stratagene) and the recombinant vector pETMAN330[16] containing the gene encoding the catalytic domain as a template. The codon for Gln91 (CAA) was replaced with AGA (Q91R) and the codon for Glu226 (GAG) with GGG (E226G). The mutant genes were sequenced to confirm the mutations. The wild type and mutant proteins were expressed and purified as described by Zhao *et al.* [16]. The protein purities were checked by SDS-PAGE[62] stained with Coomassie Brilliant Blue. The protein concentration was measured with a protein assay kit (Bio-Rad, USA), with BSA as a standard.

### Enzyme assay

The catalytic activity of the purified recombinant wide-type and mutant β-mannanases were determined using locust bean gum as substrate according to the procedure described previously[11]. Buffers, 0.05 M Na_2_HPO_4_-NaH_2_PO_4_ (pH 6.0–7.5), 0.05 M Tris-HCl (pH 7.5–8.5), 0.05 M Gly-NaOH (pH 8.5–10.5) and 0.05 M Na_2_HPO_4_-NaOH (pH 11–12) were used. Stability was determined as the residual activity after incubation at 50°C in 50 mM buffers for 1 hour.

Since the substrate is insoluble in water (and added in large quantities), the activity measurements obtained by this method can be regarded as a *k*_cat_ for insoluble locust bean gum.

### Protein Data Bank accession codes

Atomic coordinates and structure factors have been deposited with the Protein Data Bank entry code 3JUG.

## Supporting Information

Table S1 All pH-dependent activity characterized β-mannanases from GH5.
(0.10 MB DOC)

Table S2 Comparison of BSP165 MAN with other 3D structure-known β-mannanases of GH5.
(0.05 MB DOC)

Figure S1 Phylogenetic tree showing the relationship between BSP165 MAN and other characterized GH5 β-mannanases. Numbers at nodes represent the levels of bootstrap support (%) based on a neighbor-joining analysis of 1000 resampled datasets. The bar indicates a branch length equivalent to 0.2 changes per amino acid. The numbers following the enzyme abbreviations (listed in table S1) indicate the pH optima.
(0.11 MB TIF)

Figure S2 Overall structures (TIM barrel) representation of BSP165 (A), BA (B), BSP602 (C), TF (D), HJ (E) and ME MAN (F). The different views are indicated as i (top view) and ii (side view). The arrow indicates where the catalytic cleft is positioned in a (βα)_8_-barrel fold.
(1.81 MB TIF)

Figure S3 A: Stereo view of superimposition of BSP165 MAN (cyan) with BA (yellow), BSP602 (magenta) and TF (green) MAN in the catalytic site. B: Stereo view of superimposition of BSP165 MAN (cyan) with HJ MAN (pink) in the catalytic site. C: Stereo view of superimposition of BSP165 MAN (cyan) with ME MAN (gray) in the catalytic site.
(0.56 MB TIF)
