# Supplementary material for: Structural Analysis of Alkaline β-Mannanase from Alkaliphilic Bacillus sp. N16-5: Implications for Adaptation to Alkaline Conditions
Source: PLoS One. 2011 Jan 28;6(1):e14608. doi: 10.1371/journal.pone.0014608 (PMC3059134; doi:10.1371/journal.pone.0014608)
Supplement: Table S1 — All pH-dependent activity characterized β-mannanases from GH5. (0.10 MB DOC) [file pone.0014608.s001.doc]

**Table S1**

All pH-dependent activity characterized β-mannanases from GH5.

|  | Sources | GenBank | Enzyme  abbreviation | GH-family | Optimum pH | Optimum  temperature (°C) | Reference |
| --- | --- | --- | --- | --- | --- | --- | --- |
| 1 | *Bacillus* sp N16-5 | AAT06599.1 | BSP165* | GH5-8 | 9.5 | 70 | [1] |
| 2 | *Bacillus agaradhaerens* | AAN27517.1 | BA* | GH5-8 | 8-10 | 60 | [2] |
| 3 | *Bacillus* sp*. strain* JAMB-602 | BAD99527.1 | BSP602* | GH5-8 | 9 | 65 | [3] |
| 4 | *Cellvibrio japonicus* | AAO31759.1 | CJ-A | GH5-8 | 7.5-8 | NA | [4] |
| 5 | *Bacillus circulans* CGMCC 1554 | AAX87003.1 | BC1554 | GH5-8 | 7.6 | 60 | [5] |
| 6 | *Bacillus circulans* CGMCC 1416 | AAX87002.1 | BC1416 | GH5-8 | 7.6 | 58 | [6] |
| 7 | *Thermobifida fusca* KW3 | CAA06924.1 | TF* | GH5-8 | 6-8 | 80 | [7] |
| 8 | *Bacillus circulans* K-1 | BAA25878.1 | BC | GH5-8 | 6.9 | 65 | [8] |
| 9 | *Streptomyces lividans* 66 | AAA26710.2 | SL | GH5-8 | 6.8 | 58 | [9] |
| 10 | *Vibrio sp. Strain* MA-138 | BAA25188.1 | VSP-A | GH5-8 | 6.5 | 40 | [10] |
| 11 | *Caldicellulosiruptor saccharolyticus* | AAA71887.1 | CS | GH5-8 | 6-6.5 | 60-65 | [11] |
| 12 | *Caldibacillus* *cellulovorans* | AAF22274.1 | CAC | GH5-8 | 6 | 85 | [12] |
| 13 | *Thermoanaerobacterium*  *polysaccharolyticum* KM-THCJ | AAD09354.1 | TP | GH5-8 | 5.8 | 65-72 | [13] |
| 14 | *Clostridium cellulovorans* 743B | AAF06110.2 | CLC | GH5-7 | 7.0 | 45 | [14] |
| 15 | *Vibrio sp. strain* MA-138 | BAG69482.2 | VSP-C | GH5-7 | 7.0 | 50 | [15] |
| 16 | *Geobacillus stearothermophilus* MCA2184 | AAC71692.1 | GS | GH5-7 | 5.5-7.5 | NA | [16] |
| 17 | *Orpinomyces sp.* PC-2 | AAL01213.1 | OSP | GH5-7 | 4.6-7.2 | 50 | [17] |
| 18 | *Cellvibrio japonicus* | AAO31761.1 | CJ-C | GH5-7 | 5-6.5 | NA | [4] |
| 19 | *Emericella nidulans* FGSC A4 | ABF50863.1 | EN | GH5-7 | 5.5 | 52 | [18] |
| 20 | *Armillariella tabescens* EJLY2098 | ABB88954.1 | AT | GH5-7 | 5.5 | 60 | [19] |
| 21 | *Phanerochaete chrysosporium* RP78 | ABG79370.1 | PC | GH5-7 | 4-6 | 72 | [20] |
| 22 | *Aspergillus aculeatus* KSM 510 | AAA67426.1 | AA | GH5-7 | 5.0 | 60-70 | [21] |
| 23 | *Aspergillus fumigatus* IMI385708 | ACH58410.1 | AF1 | GH5-7 | 4.5 | 60 | [22] |
| 24 | *Aspergillus fumigatus* IMI385708 | ACH58411.1 | AF2 | GH5-7 | 4.5 | 60 | [22] |
| 25 | *Hypocrea jecorina* RUTC-30 | AAA34208.1 | HJ* | GH5-7 | 3.5-4 | 70 | [23] |
| 26 | *Aspergillus sulphureus* | ABC59553.1 | AS | GH5-7 | 2.4 | 50 | [24] |
| 27 | *Bispora sp.* MEY-1 | ACH56965.1 | BISP | GH5-7 | 1-1.5 | 65 | [25] |
| 28 | *Haliotis discus hannai* | BAE78456.1 | HDH | GH5-10 | 7.5 | 45 | [26] |
| 29 | *Mytilus edulis* | CAC81056.1 | ME* | GH5-10 | 5.2 | 50 | [27] |
| 30 | *Cryptopygus antarcticus* | ABV68808.1 | CA | GH5-10 | 3.5 | 30 | [28] |

The temperature optima for CJ-A, GS, CJ-C are not available. NA = not available. * 3D structure has been solved.

References

1. Ma Y, Xue Y, Dou Y, Xu Z, Tao W, et al. (2004) Characterization and gene cloning of a novel beta-mannanase from alkaliphilic *Bacillus* sp. N16-5. Extremophiles 8: 447-454.

2. Kauppinen MS, Schülein M, Schnorr K, Andersen LN, Bjørnvad ME (2003) Mannanases. United States: Novozymes, A/s (Bagsvaerd, DK).

3. Akita M, Takeda N, Hirasawa K, Sakai H, Kawamoto M, et al. (2004) Crystallization and preliminary X-ray study of alkaline mannanase from an alkaliphilic *Bacillus* isolate. Acta Crystallographica, Section D: Biological Crystallography 60: 1490-1492.

4. Hogg D, Pell G, Dupree P, Goubet F, Martin-Orue SM, et al. (2003) The modular architecture of *Cellvibrio japonicus* mannanases in glycoside hydrolase families 5 and 26 points to differences in their role in mannan degradation. Biochemical Journal 371: 1027-1043.

5. Yang P, Li Y, Wang Y, Meng K, Luo H, et al. (2008) A novel beta-mannanase with high specific activity from *Bacillus circulans* CGMCC1554: gene cloning, expression and enzymatic characterization. Applied Biochemistry and Biotechnology: 1559-0291 (Electronic).

6. Li Y, Yang P, Meng K, Wang Y, Luo H, et al. (2008) Gene cloning, expression, and characterization of a novel beta-mannanase from *Bacillus circulans* CGMCC 1416. Journal of Microbiology and Biotechnology 18: 160-166.

7. Hilge M, Gloor SM, Rypniewski W, Sauer O, Heightman TD, et al. (1998) High-resolution native and complex structures of thermostable beta-mannanase from *Thermomonospora fusca*  substrate specificity in glycosyl hydrolase family 5. Structure 6: 1433-1444.

8. Yoshida S, Sako Y, Uchida A (1998) Cloning, sequence analysis, and expression in *Escherichia coli* of a gene coding for an enzyme from *Bacillus circulans* K-1 that degrades guar gum. Bioscience, Biotechnology, and Biochemistry 62: 514-520.

9. Arcand N, Kluepfel D, Paradis FW, Morosoli R, Shareck F (1993) β-mannanase of *Streptomyces lividans* 66: cloning and DNA sequence of the manA gene and characterization of the enzyme. Biochemical Journal 290 ( Pt 3): 857-863.

10. Tamaru Y, Araki T, Amagoi H, Mori H, Morishita T (1995) Purification and characterization of an extracellular beta-1,4-mannanase from a marine bacterium, *Vibrio* sp. strain MA-138. Applied and Environmental Microbiology 61: 4454-4458.

11. Gibbs MD, Elinder AU, Reeves RA, Bergquist PL (1996) Sequencing, cloning and expression of a beta-1,4-mannanase gene, manA, from the extremely thermophilic anaerobic bacterium, *Caldicellulosiruptor* Rt8B.4. FEMS Microbiology Letters 141: 37-43.

12. Sunna A, Gibbs MD, Chin CW, Nelson PJ, Bergquist PL (2000) A gene encoding a novel multidomain beta-1,4-mannanase from *Caldibacillus cellulovorans* and action of the recombinant enzyme on kraft pulp. Applied and Environmental Microbiology 66: 664-670.

13. Cann IK, Kocherginskaya S, King MR, White BA, Mackie RI (1999) Molecular cloning, sequencing, and expression of a novel multidomain mannanase gene from *Thermoanaerobacterium polysaccharolyticum*. Journal of Bacteriology 181: 1643-1651.

14. Tamaru Y, Doi RH (2000) The engL gene cluster of *Clostridium cellulovorans* contains a gene for cellulosomal manA. Journal of Bacteriology 182: 244-247.

15. Tanaka M, Umemoto Y, Okamura H, Nakano D, Tamaru Y, et al. (2009) Cloning and characterization of a β-1,4-mannanase 5C possessing a family 27 carbohydrate-binding module from a marine bacterium, *Vibrio* sp. strain MA-138. Bioscience, Biotechnology, and Biochemistry 73: 109-116.

16. Talbot G, Sygusch J (1990) Purification and characterization of thermostable β-mannanase and α-galactosidase from *Bacillus stearothermophilus*. Applied and Environmental Microbiology 56: 3505-3510.

17. Ximenes EA, Chen H, Kataeva IA, Cotta MA, Felix CR, et al. (2005) A mannanase, ManA, of the polycentric anaerobic fungus *Orpinomyces* sp. strain PC-2 has carbohydrate binding and docking modules. Canadian Journal of Microbiology 51: 559-568.

18. Bauer S, Vasu P, Persson S, Mort AJ, Somerville CR (2006) Development and application of a suite of polysaccharide-degrading enzymes for analyzing plant cell walls. Proceedings of the National Academy of Sciences of the United States of America 103: 11417-11422.

19. Wang Y, Wong A, Huang X, Liu D, Yao D (2009) Cloning, expression and characterization of mannanase from *Armillariella tabescens* EJLY2098 in *Pichia pastoris*. Sheng Wu Gong Cheng Xue Bao 25: 920-926.

20. Benech R-O, Li X, Patton D, Powlowski J, Storms R, et al. (2007) Recombinant expression, characterization, and pulp prebleaching property of a *Phanerochaete chrysosporium* endo-β-1,4-mannanase. Enzyme and Microbial Technology 41: 740-747.

21. Christgau S, Kauppinen S, Vind J, Kofod LV, Dalboge H (1994) Expression cloning, purification and characterization of a beta-1,4-mannanase from *Aspergillus aculeatus*. Biochemistry and Molecular Biology International 33: 917-925.

22. Puchart V, Vrsanska M, Svoboda P, Pohl J, Ogel ZB, et al. (2004) Purification and characterization of two forms of endo-beta-1,4-mannanase from a thermotolerant fungus, *Aspergillus fumigatus* IMI 385708 (formerly *Thermomyces lanuginosus* IMI 158749). Biochimica et Biophysica Acta 1674: 239-250.

23. Stalbrand H, Siika-aho M, Tenkanen M, Viikari L (1993) Purification and characterization of two β-mannanases from *Trichoderma reesei*. Journal of Biotechnology 29: 229-242.

24. Chen X, Cao Y, Ding Y, Lu W, Li D (2007) Cloning, functional expression and characterization of *Aspergillus sulphureus* beta-mannanase in *Pichia pastoris*. Journal of Biotechnology 128: 452-461.

25. Luo H, Wang Y, Wang H, Yang J, Yang Y, et al. (2009) A novel highly acidic β-mannanase from the acidophilic fungus *Bispora* sp. MEY-1: gene cloning and overexpression in *Pichia pastoris*. Applied Microbiology and Biotechnology 82: 453-461.

26. Ootsuka S, Saga N, Suzuki K, Inoue A, Ojima T (2006) Isolation and cloning of an endo-beta-1,4-mannanase from Pacific abalone *Haliotis discus hannai*. Journal of Biotechnology 125: 269-280.

27. Xu B, Hagglund P, Stalbrand H, Janson JC (2002) endo-beta-1,4-Mannanases from blue mussel, *Mytilus edulis*: purification, characterization, and mode of action. Journal of Biotechnology 92: 267-277.

28. Song JM, Nam KW, Kang SG, Kim CG, Kwon ST, et al. (2008) Molecular cloning and characterization of a novel cold-active beta-1,4-D-mannanase from the Antarctic springtail, *Cryptopygus antarcticus*. Comparative Biochemistry and Physiology Part B, Biochemistry and Molecular Biology 151: 32-40.
