# Supplementary material for: Structural Analysis of Alkaline β-Mannanase from Alkaliphilic Bacillus sp. N16-5: Implications for Adaptation to Alkaline Conditions
Source: PLoS One. 2011 Jan 28;6(1):e14608. doi: 10.1371/journal.pone.0014608 (PMC3059134; doi:10.1371/journal.pone.0014608)
Supplement: Table S2 — Comparison of BSP165 MAN with other 3D structure-known β-mannanases of GH5. (0.05 MB DOC) [file pone.0014608.s002.doc]

# Table S2

Comparison of BSP165 MAN with other 3D structure-known β-mannanases of GH5.

| Parameters | BSP165 | BA | BSP602 | TF | ME | HJ |
| --- | --- | --- | --- | --- | --- | --- |
| Optimum pH | 9.5 | 8-10 | 9 | 6-8 | 5.2 | 3.5-4 |
| Optimum temperature (K) | 343 | 333 | 338 | 353 | 323 | 343 |
| amino acids calculated for | 295 | 296 | 302 | 298 | 353 | 344 |
| Amino acid identity (%) | - | 97 | 78 | 45 | 17 | 13 |
| PDB code | 3jug | 2whj | 1wky | 1bqc | 2c0h | 1qno |
| Z | - | 57.4 | 55.4 | 47.1 | 19.3 | 22.2 |
| Rmsd | - | 0.3 | 0.7 | 1.3 | 3.1 | 2.8 |
| α-helix (%) | 34.1 | 35.1 | 32.5 | 34.6 | 29.7 | 34.9 |
| β-strand (%) | 16.3 | 16.2 | 17.2 | 16.1 | 17.8 | 16.6 |
| secondary structure | 50.4 | 51.3 | 49.7 | 50.7 | 47.6 | 51.5 |
| Hydrogen bonds(%) [DSSP] | 73.5 | 70.9 | 71.8 | 71.4 | 70.3 | 68.0 |
| Ion Pairs (%) | 7.12 | 7.09 | 5.30 | 6.71 | 4.25 | 6.40 |
| Compactness coefficient | 1.61 | 1.61 | 1.60 | 1.60 | 1.71 | 1.73 |

Z: Z-score (statistical significance) of the best domain-domain alignment

Compactness coefficient: the ratio of the accessible surface area of a protein to that of the ideal sphere of the same volume
