# Supplementary figures and images for: Structural Analysis of Alkaline β-Mannanase from Alkaliphilic Bacillus sp. N16-5: Implications for Adaptation to Alkaline Conditions
Source: PLoS One. 2011 Jan 28;6(1):e14608. doi: 10.1371/journal.pone.0014608 (PMC3059134; doi:10.1371/journal.pone.0014608)

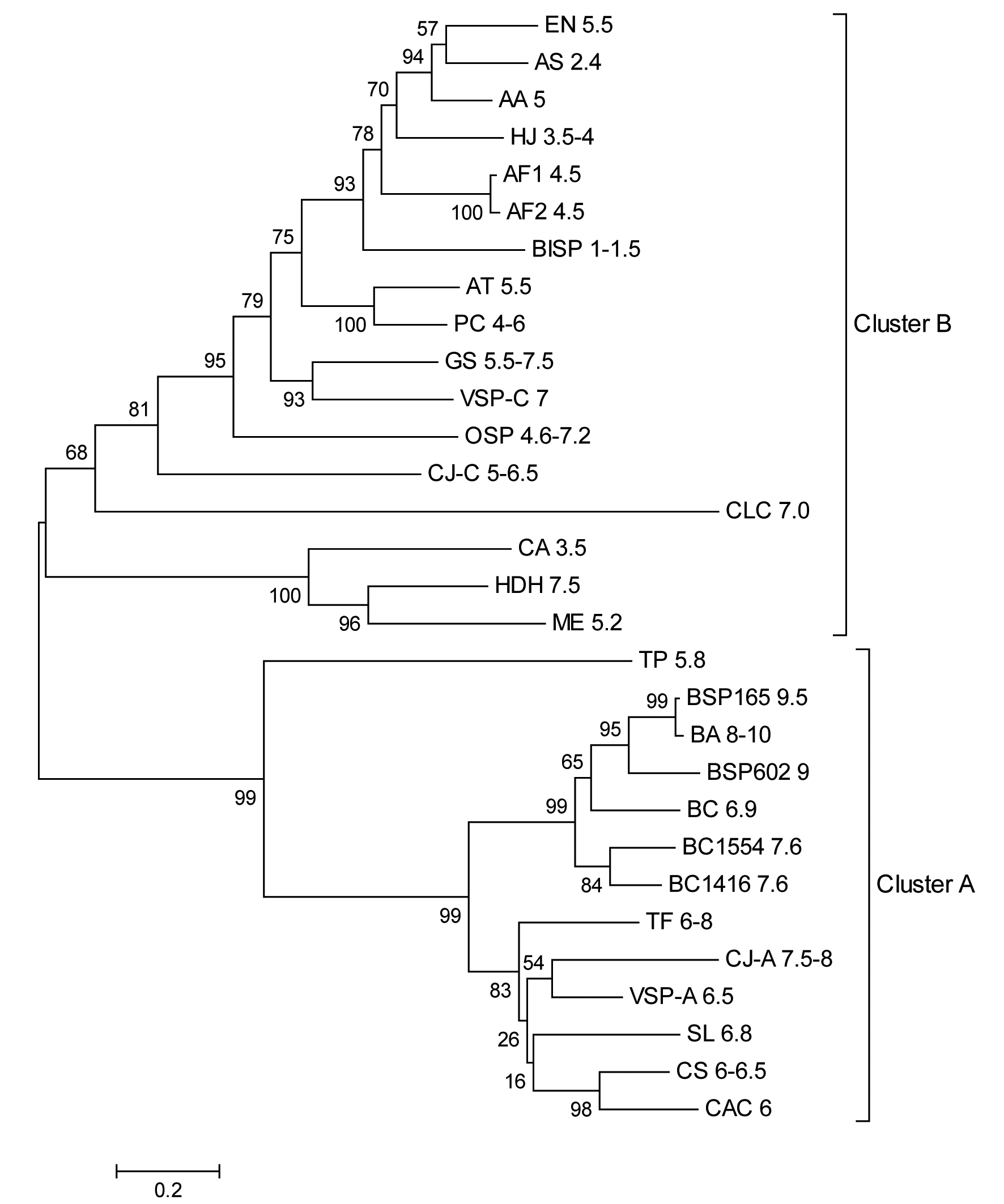

Supplement: Figure S1 — Phylogenetic tree showing the relationship between BSP165 MAN and other characterized GH5 β-mannanases. Numbers at nodes represent the levels of bootstrap support (%) based on a neighbor-joining analysis of 1000 resampled datasets. The bar indicates a branch length equivalent to 0.2 changes per amino acid. The numbers following the enzyme abbreviations (listed in table S1) indicate the pH optima. (0.11 MB TIF) [file pone.0014608.s003.tif]

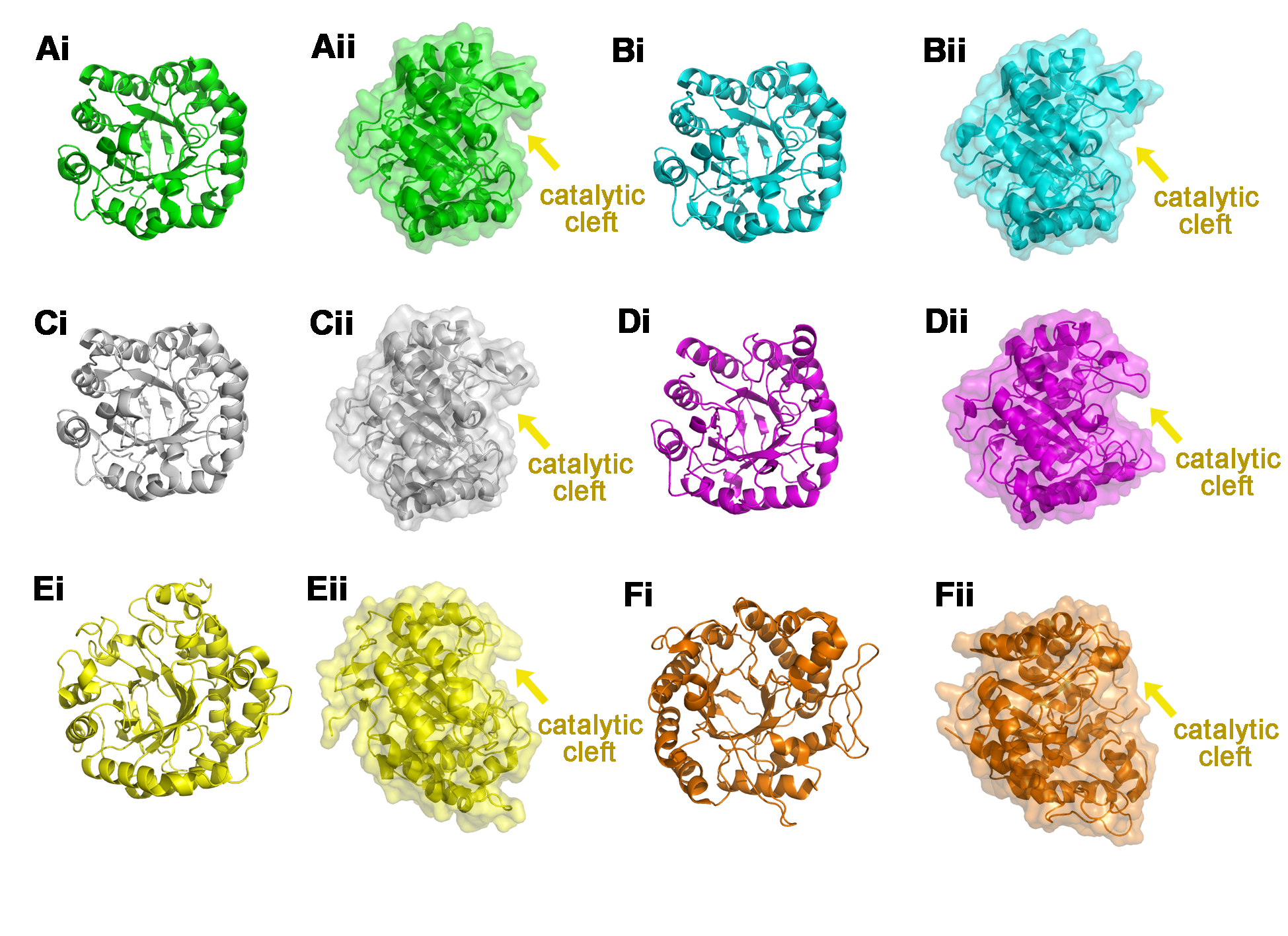

Supplement: Figure S2 — Overall structures (TIM barrel) representation of BSP165 (A), BA (B), BSP602 (C), TF (D), HJ (E) and ME MAN (F). The different views are indicated as i (top view) and ii (side view). The arrow indicates where the catalytic cleft is positioned in a (βα)8-barrel fold. (1.81 MB TIF) [file pone.0014608.s004.tif]

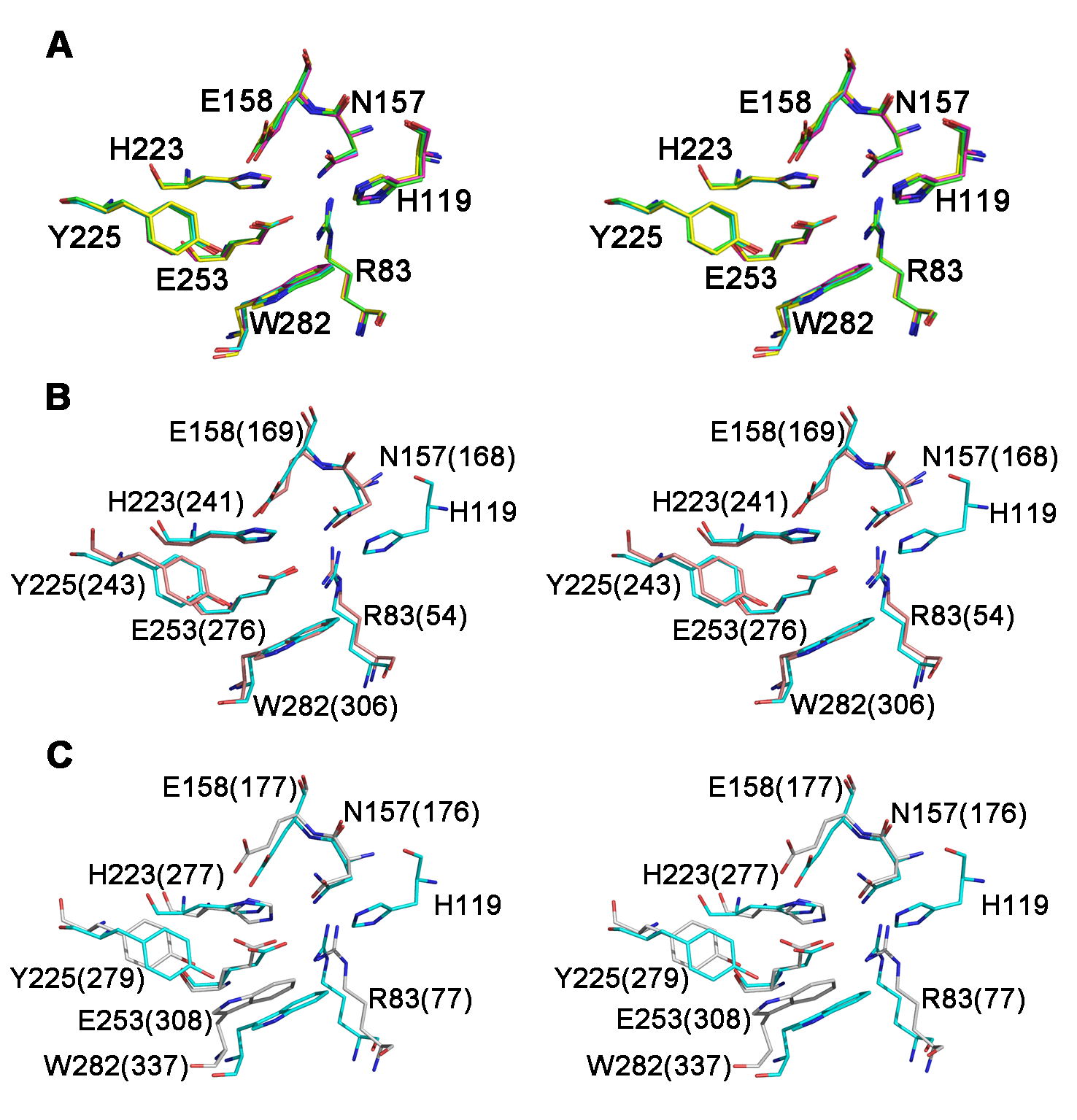

Supplement: Figure S3 — A: Stereo view of superimposition of BSP165 MAN (cyan) with BA (yellow), BSP602 (magenta) and TF (green) MAN in the catalytic site. B: Stereo view of superimposition of BSP165 MAN (cyan) with HJ MAN (pink) in the catalytic site. C: Stereo view of superimposition of BSP165 MAN (cyan) with ME MAN (gray) in the catalytic site. (0.56 MB TIF) [file pone.0014608.s005.tif]
